# Population structure and gene flow of the tropical seagrass, *Syringodium filiforme*, in the Florida Keys and subtropical Atlantic region

**DOI:** 10.1371/journal.pone.0203644

**Published:** 2018-09-05

**Authors:** Alexandra L. Bijak, Kor-jent van Dijk, Michelle Waycott

**Affiliations:** 1 Department of Environmental Sciences, University of Virginia, Charlottesville, Virginia, United States of America; 2 School of Biological Sciences, Environment Institute, Australian Centre for Evolutionary Biology and Biodiversity, University of Adelaide, Adelaide, South Australia, Australia; 3 State Herbarium of South Australia, Department of Environment, Water and Natural Resources, Adelaide, South Australia, Australia; Department of Agriculture and Water Resources, AUSTRALIA

## Abstract

Evaluating genetic diversity of seagrasses provides insight into reproductive mode and adaptation potential, and is therefore integral to broader conservation strategies for coastal ecosystems. In this study, we assessed genetic diversity, population structure and gene flow in an opportunistic seagrass, *Syringodium filiforme*, in the Florida Keys and subtropical Atlantic region. We used microsatellite markers to analyze 20 populations throughout the Florida Keys, South Florida, Bermuda and the Bahamas primarily to understand how genetic diversity of *S*. *filiforme* partitions across the Florida Keys archipelago. We found low allelic diversity within populations, detecting 35–106 alleles across all populations, and in some instances moderately high clonal diversity (*R* = 0.04–0.62). There was significant genetic differentiation between Atlantic and Gulf of Mexico (Gulf) populations (*F*_*ST*_ = 0.109 ± 0.027, *p-value* = 0.001) and evidence of population structure based on cluster assignment, dividing the region into two major genetic demes. We observed asymmetric patterns in gene flow, with a few instances in which there was higher than expected gene flow from Atlantic to Gulf populations. In South Florida, clustering into Gulf and Atlantic groups indicate dispersal in *S*. *filiforme* may be limited by historical or contemporary geographic and hydrologic barriers, though genetic admixture between populations suggests exchange may occur between narrow channels in the Florida Keys, or has occurred through other mechanisms in recent evolutionary history, maintaining regional connectivity. The variable genotypic diversity, low genetic diversity and evidence of population structure observed in populations of *S*. *filiforme* resemble the population genetics expected for a colonizer species.

## Introduction

Genetic diversity is paramount to the long-term survival of populations, as genetic variation provides the basis for adaptation to environmental change via natural selection and confers short-term fitness advantages at the population level. Population structure and gene flow, which describe the level of genetic differentiation and connectivity between populations, are important components and drivers of genetic diversity. Quantifying genetic diversity within and among natural populations enhances conservation efforts because genetic patterns are difficult to predict given the complex suite of environmental and biological factors that contribute to genetic diversity and population structure [1]. In seagrass ecosystems, genetic diversity is of particular concern because within-species diversity may replace the functional role of species diversity due to the limited number of species present in seagrass communities [2]. Within-species diversity is also important to the short-term population persistence of seagrasses as genetically diverse assemblages of multiple unique genotypes (or clones) promote greater resistance and faster recovery following disturbance [3,4]. Successional stage, an aggregate category based on multiple traits, provides an ecological lens to assess broad patterns in plant genetic diversity and population structure. Examining population genetics within the context of ecological succession aids in identifying traits that foster resilience in populations of foundational taxa such as seagrasses, and thereby the ecosystems they support.

Theory suggests early successional species are expected to have diminished genetic diversity due to founder effects and to develop strong population structure due to limited gene flow, while later successional species are typified by greater standing genetic diversity and weaker population structure [5]. In terrestrial ecosystems, long-lived woody species tend to have more genetic diversity within populations and less variation between populations based on allozyme studies [6] in congruence with expectations, though patterns in terrestrial pioneering species are less clear. Populations of an early successional species, *Silene dioica*, in the Gulf of Bothnia show strong differentiation when the supply of colonists is limited [7], while other European early colonizing plant species have higher than expected genetic diversity within populations and low genetic differentiation between populations [8,9]. The relationship between successional status and population genetics in seagrasses, however, has not been thoroughly explored.

Seagrasses present an opportunity to study environmental and ecological determinants of genetic diversity because they comprise a globally distributed, paraphyletic taxon that has evolved from up to four independent lineages [10] and represents a spectrum of life history strategies. Analyses of diversity, population structure and gene flow can reveal biological and physical phenomena that promote or deter the exchange of genetic material across populations. Species biological traits such as breeding system, pollination mechanisms and dispersal capability strongly influence genetic diversity as measured by genotypic diversity, gene copy (or allele) diversity and heterozygosity [11]. The capability to propagate through horizontal rhizome expansion and reproduction by seed has led to early notions that seagrasses are predominantly clonal and therefore lack genetic diversity [12,13]. The development of high-resolution markers prompted studies that have countered this expectation by detecting higher genetic diversity than initially reported for several seagrass species [14], generating questions regarding the role of dispersal and sexual reproduction in shaping seagrass population genetics. Environmental conditions, such as water quality, prevailing winds and local water movement, contribute to fine-scale population genetic structure in seagrasses [15,16], while geographic history, including glaciation and continental drift, in conjunction with modern gene flow patterns influenced by oceanic hydrology, determine genetic connectivity at broader spatial scales [17]. In this study, we described the genetic diversity, population structure and gene flow of the opportunistic seagrass, *Syringodium filiforme*, in the Florida Keys and subtropical Atlantic region.

*S*. *filiforme* is widely distributed throughout the western tropical and subtropical Atlantic Ocean in shallow coastal and back reef environments [18,19], and is a common species in seagrass meadows that cover as many as 17,629 km^2^ of South Florida coastline [20]. These meadows support marine food webs including epiphytic algae to large grazers, deliver ecosystem services by stabilizing coastal sediments and improving local water quality, and have recently been recognized for their role in carbon storage [21,22]. Seagrasses in this region are threatened by local impacts related to water quality such as sedimentation and nutrient over-enrichment [23–25] and have experienced substantial die-offs within the past several decades, notably in Florida Bay [26–29] and Tampa Bay [30]. In order to understand the full impact of environmental decline and perturbations in seagrass ecosystems, evaluating existing levels of genetic diversity, population structure and gene flow is essential. Sampling locations for this study spanned across tens of kilometers in the focal area of the Florida Keys and South Florida, but also included remote populations in Bermuda and the Bahamas at distances of hundreds to thousands of kilometers apart in order to compare diversity in South Florida populations to diversity across more distant populations.

*S*. *filiforme* generally dominates early successional meadows because it has relatively high horizontal rhizome elongation rates [31] and tolerates sediment conditions that are less favorable to other dominant seagrasses, but is also present in the climax state [32]. These traits enable *S*. *filiforme* to quickly colonize bare areas through clonal propagation, but also through seed and vegetative fragment dispersal, especially following disturbance [33]. Based on the ability to colonize, reproduce by seed, and generate and maintain substantial biomass, Kilminster et al. [34] categorized *Syringodium* species as opportunistic, exhibiting a mixture of life history traits found in both colonizing and persistent species. Previous studies have characterized the genetic diversity of other common South Florida seagrasses, *Thalassia testudinum* and *Halodule wrightii*. In line with theory, *T*. *testudinum*, a late successional seagrass species, exhibited high genetic diversity within populations and weak genetic structure in Florida Bay and the Lower Keys (regions within the Florida Keys are typically described on a north-south basis as Upper, Middle and Lower Keys) [35–37]. As expected for an early colonizer and opportunistic species, most of the genetic variation in *H*. *wrightii* partitioned among populations rather than within populations in a study focused on the Gulf of Mexico and Florida Bay [38]. We hypothesized *S*. *filiforme* would exhibit genetic diversity and population structure patterns similar to those expected for colonizer species, and would therefore reveal high clonality, low genetic diversity and strong differentiation between populations throughout the Florida Keys and wider study area.

South Florida coastal waters exhibit particularly complex hydrology, especially around the Florida Keys, an archipelago that spans 350 km from the South Florida mainland to Key West, separating the Gulf of Mexico and Florida Bay from the Atlantic Ocean [39]. The distribution of *S*. *filiforme* across the Florida Keys ranges from marginal and patchy in northeastern Florida Bay, to sparse in offshore intermixed beds on the Atlantic Ocean (hereafter referred to as Atlantic) side, to dense, monospecific stands along the Middle and Lower Keys on the Gulf of Mexico (hereafter referred to as Gulf) side [40,41]. The division created by the Florida Keys archipelago separates geographically proximal *S*. *filiforme* populations in the Atlantic and Gulf basins, leading us to predict these basins host genetically distinct populations due to limited opportunity for propagule exchange and gene flow across a physical barrier. We expected the Bahamas population to have high genetic connectivity with the Florida populations because of its relative proximity, but the Bermuda population to be genetically distinct from the Florida populations due to its geographic isolation.

In this study, we used species-specific microsatellite loci to assess genetic diversity, population genetic structure and connectivity via gene flow in *S*. *filiforme* across the Florida Keys and subtropical Atlantic region. We examined 1) whether clonality varies within 100s of m^2^ and 1000s of m^2^ spatial scales; 2) the relative amounts of genetic diversity present within individuals and populations of *S*. *filiforme*; 3) the degree to which genetic differentiation among populations results in population structure; and 4) whether there are patterns in the magnitude and direction of gene flow between populations.

## Methods

### Sample collection

**Fig 1:**
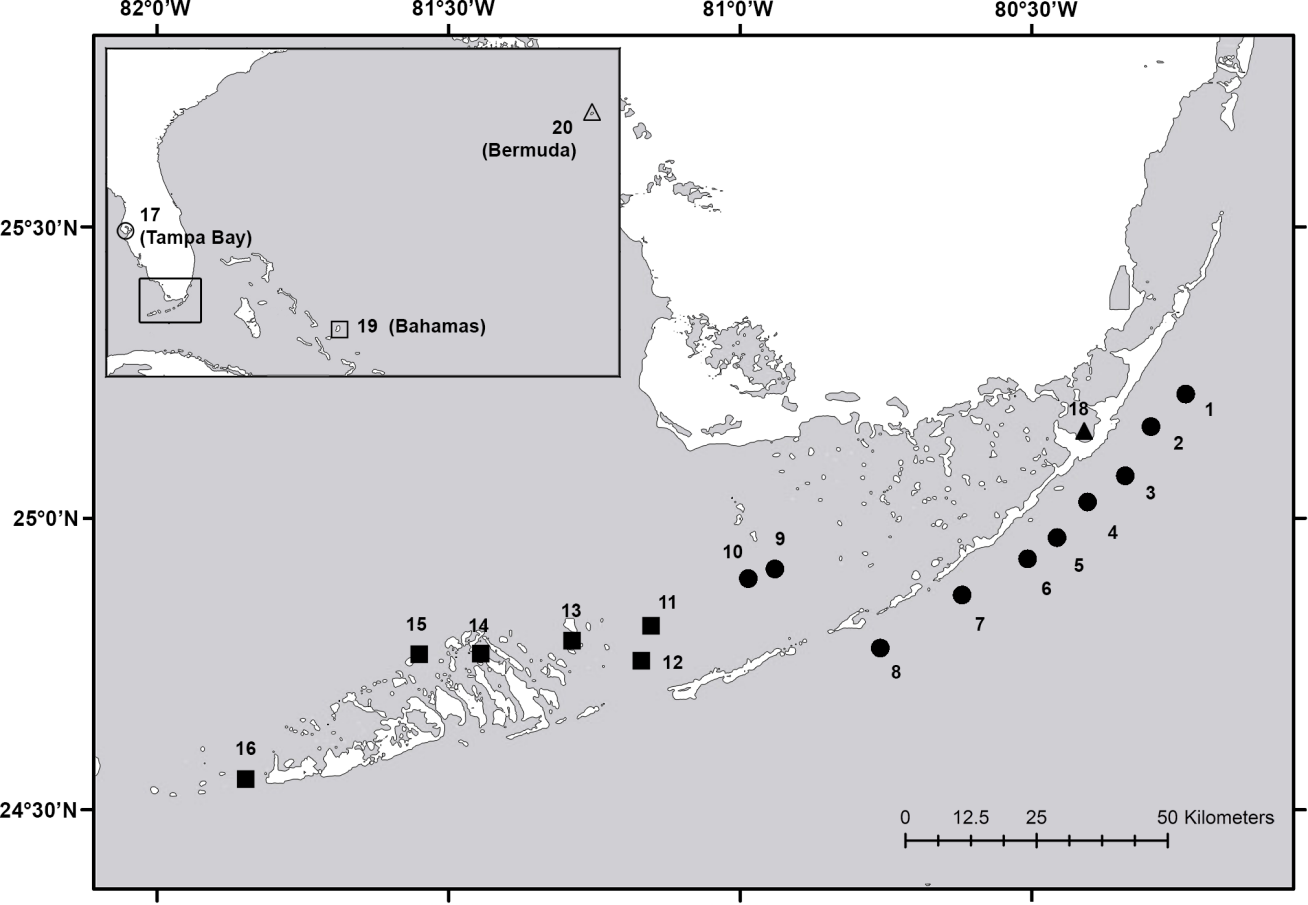
Map of study area and sampling locations. The inset map shows the relative positions of the Florida Keys, Tampa Bay, the Bahamas and Bermuda. The main map shows the positions of the Florida Keys sampling locations. Site numbers are displayed to minimize text in the figure (corresponding site names are available in Table 1). Sampling methodology is represented by shape in both the main and inset maps (sampling area of ~ 2,500 m^2^: circle; sampling area of ~ 500 m^2^: square; Florida Bay and Bermuda–composite sampling areas of ~ 70 m^2^: triangle).

We sampled a total of 20 meadows, hereafter termed populations, in South Florida, the Bahamas and Bermuda (Fig 1; see S1 Table for site GPS coordinates) following three sampling designs over the summers of 2014 and 2015. In 2014, we sampled within a ~ 2,500 m^2^ area to estimate clonal extent. After detecting unique genotypes within meters of each other, we reduced the sampling area in 2015 and modified the sampling area for Florida Bay and Bermuda in order to accommodate for greater patchiness of *S*. *filiforme* meadows. Genetic data collected with uneven but similar sampling schemes are comparable when using unique genotypes for regional analyses of genetic diversity and population structure [42], assuming the alleles detected are representative of the areas sampled [43,44]. The use of three sampling approaches did not allow for direct comparison of genotypic diversity across sampling designs; however, the primary goal of this study was to determine allelic diversity in order to evaluate population structure and regional connectivity, not to determine fine-scale population structure or the spatial distribution of clones.

In 2014, we sampled eight populations in the Upper and Middle Keys on the Atlantic side, two populations on the Gulf side (Sprigger and Sluiceway) and a single population in Tampa Bay. In 2015, we sampled six populations in the Middle and Lower Keys on the Gulf side and one population in the northeastern portion of Florida Bay. Additionally in 2015, we sampled single populations in San Salvador, the Bahamas and Bailey’s Bay, Bermuda. Leaves of at least 50 individual *S*. *filiforme* ramets were randomly collected within a ~ 2,500 m^2^ sampling area, spaced 5 m apart, for the 2014 collection, and within a ~ 500 m^2^ sampling area, spaced 1.5 m apart, for most of the 2015 collection. At the Florida Bay and Bermuda sites where the distribution of *S*. *filiforme* was limited, six and five smaller areas (spaced < 1 km) were sampled, respectively. Within each area, 24 leaves were collected from ramets (spaced 1.5 m apart) in a ~ 70 m^2^ sampling area.

### Ethics statement

Permits were required for sample collection in the Lower Florida Keys (Florida Keys National Marine Sanctuary) and Florida Bay (Everglades National Park) in 2015 because sediment was collected in addition to seagrass plant tissue for supplemental analyses; sampling in these areas was conducted under FKNMS-2015-085 and EVER-2013-SCI-0058, respectively. Sampling in Bermuda was conducted under the Bermuda Dept. of Conservation Services License no. 15-04-16-22.

### Genotyping

Total genomic DNA was extracted from the samples collected in 2014 using a DNeasy™ Plant Kit (QIAGEN) according to the manufacturer’s instructions. Extracted DNA was quantified on a Qubit® 2.0 Fluorometer (Invitrogen). Samples collected in 2015 were sent to the University of Wisconsin Biotechnology (University of Wisconsin, Wisconsin, USA) for extraction and quantification. DNA was extracted from 40–50 mg of dried leaf tissue using the CTAB method as described in Saghai-Maroof et al. with minimal modification [45]. Following elution, a final DNA cleaning step was performed using a 1.5:1 by volume ratio of Axygen Clean-Seq beads (Corning Life Sciences, Corning, NY, USA) to extracted DNA to remove any remaining inhibitory compounds in the sample. DNA was quantified using Quant-IT PicoGreen fluorescent dye (Thermo Fisher, Waltham, MA, USA). All extracted DNA was diluted to a concentration of ~5ng μL^-1^. For some samples, DNA extraction was unsuccessful due to the poor tissue quality of senescing seagrass leaves, reducing the sample size for several sites.

A total of 17 microsatellite loci were amplified using fluorescently labeled primers [46]. PCR was conducted in three PCR multiplex panels using a Type-it® Microsatellite Multiplex PCR Kit (QIAGEN) in 10 μreactions with 0.5 μL of 2 μM primer mix and 1 μL of diluted template DNA. PCR conditions were set to the manufacturer’s optimized cycling conditions (QIAGEN). PCR products were sequenced on a capillary-based 3730xl DNA Analyzer (Applied Biosystems) with an internal ET-ROX 500 size standard at the Georgia Genomics Facility (University of Georgia, Georgia, USA). Fragment lengths for each locus were determined using the Geneious v7.1.9 (Biomatters Ltd.) and microsatellite plugin [v1.4.0]. Verification samples from 2014 were included in 2015 PCR and sequencing steps to assess the reproducibility of our methods. Approximately 32% of the verification sample loci either did not successfully amplify during PCR or did not produce microsatellite peaks when sequenced, likely due to pipetting error. When verification samples were successfully amplified and sequenced, discrepancies between microsatellite peaks in 2014 and 2015 occurred for less than 3% of samples.

### Within-population genetic diversity

The number of unique multi-locus genotypes (MLGs), *G*, the probability individuals sharing the same genotype were derived via separate sexual events, (*P*_sex_), and the probability of clonal identity, (*P*_gen_), were estimated for each population using Genclone version 2.0 [47,48]. Unique MLGs were identified under the assumption that scoring error and somatic mutation rates were negligible (genotypes with a single allele difference were considered distinct). We tested for the presence of null alleles across all populations using ML-Null Freq with 100,000 randomizations [49]. Genotypic richness, *R*, the proportion of genetically distinct individuals (or genets) in the population, was calculated as *R = (G-1)/(N-1)* [50].

For the remainder of population genetic analyses, replicate MLGs were removed from the dataset to avoid allele frequency bias due to the presence of clones. The total number of alleles, *A*, average number of alleles per locus, *N*_*A*_, and average allelic richness per locus standardized by smallest sample size, *A*_*R*_, were calculated using the ‘diveRsity’ package [51] in R [52]. For each population, observed heterozygosity (*H*_*o*_), expected heterozygosity (*H*_*e*_), and deviation from Hardy-Weinberg equilibrium as measured by the inbreeding coefficient, *F*_*IS*_, were calculated in Genalex version 6.5 [53,54]. We calculated linkage disequilibrium for each population using log-likelihood tests in Genepop version 4.2 [55,56] and determined significance using a sequential Bonferroni correction to account for multiple comparisons.

### Genetic differentiation between populations

An analysis of molecular variance (AMOVA) was performed first on all populations to assess overall genetic differentiation, and again with only populations bordering the archipelago (populations with numeric codes 1–16 in Fig 1) in a nested design to evaluate differentiation between the Gulf and Atlantic populations, following the assumptions of the Infinite Allele Model in Genodive [57]. Standard deviations for AMOVA F-statistics were calculated by jackknife resampling over loci, and 999 permutation tests were used to assess significance. Fixation indices Weir and Cockerham’s *F*_*ST*_ [58] and Jost’s *D* [59] were calculated for all possible pairwise population combinations using the ‘diveRsity’ package in R. Statistical significance was determined by 95% confidence intervals derived from bias corrected bootstrapping. Principal components analysis (PCA) was performed in Genodive using a covariance matrix based on individual allele frequencies to determine whether geographically proximal samples exhibit similar allele frequencies, but without the assumption of hierarchical genetic structure.

### Population structure and gene flow

To determine the most likely number of population clusters, K, population assignment utilizing a Bayesian approach was performed in the genetic software program structure [60]. Admixture was specified in the model, allowing genotypes to show membership to more than one cluster. The correlated allele frequency model was selected and sampling locations were not used as priors in the analysis. Model parameters were set to K = 1–20, with 10 iterations run for each K, and an initial burn-in period of 100,000 iterations (sufficient for α, *F*_ST_ to converge) followed by 1,000,000 Markov Chain Monte Carlo repetitions. The most likely number of population clusters was determined by the ad hoc quantity, Δ*K* [61]. Complementary software programs, clumpak [62] were used for downstream processing, and distruct was used for visual representation of the results [63].

Average total migration, *N*_*m*_, was estimated using *F*_*ST*_ [64] and rare alleles methods [65] in Genalex and Genepop, respectively. Additionally, pairwise relative migration rates were estimated using Alcala’s *Nm* [66] and directionality of differentiation was estimated according to methods developed by Sundqvist et al. [67] using ‘diveRsity’ in R.

## Results

### Within-population genetic diversity

For most populations, *P*_*gen*_ ranged from 3.3 x 10^−8^ to 7.0 x 10^−3^, indicating there was a low probability of generating the observed genotypes under Hardy-Weinberg Equilibrium conditions. *P*_*sex*_ ranged from 1.7 x 10^−7^ to 2.0 x 10^−3^, though there were higher values for *P*_*sex*_ in the following Florida populations: Crane (0.094), Key West (0.15), Tampa Bay (0.063), and Florida Bay (0.19). The few instances in which *P*_*sex*_ exceeded 0.05 occurred in populations dominated by few clones, thereby inflating *P*_*sex*,_ and were unlikely to have greatly impacted the accuracy of heterozygosity estimates and other statistical analyses performed. The 37 instances (of the total 2,720 pairwise comparisons) in which linkage disequilibrium was significant after a Bonferroni correction was applied (*p-value* < 0.003) were also unlikely to affect subsequent population genetic analyses.

Genotypic richness was highly variable among populations, ranging from 0.04 to 0.62 (Table 1). Genotypic richness for Florida Keys populations sampled in 2014 and 2015 ranged from 0.37 to 0.62 and from 0.05 to 0.43, respectively. Genotypic richness values may have been overestimated because we did not account for scoring error or somatic mutation when identifying unique MLGs. The total number of alleles ranged from 35 to 106 and the average number of alleles per locus ranged from 2.06 to 6.24. Once adjusted for sample size, allelic richness was similar across all populations, ranging from 2.03 to 2.56. Observed heterozygosity ranged from 0.21 to 0.51, and expected heterozygosity ranged from 0.18 to 0.44. Deviation from Hardy-Weinberg conditions was detected in nine populations (*p*-*value* < 0.05), most of which exhibited negative inbreeding coefficients. We found no significant effect of null alleles, except in populations with few genets (G ≤ 10) and loci for which all samples were homozygous for the same allele, or fixed. Excluding populations with few genets and loci with fixed alleles, the mean per locus significance of heterozygote deficiency due to null alleles across populations ranged from 0.276 to 0.827.

**Table 1:** Summary genetic statistics for all populations.

Population		N	G	R	A	N_A_	A_R_	H_o_	H_e_	F_IS_
**1**	Carysfort	48	19	0.38	98	5.76	2.53	0.51 ± 0.08	0.43 ± 0.07	**-0.16 ± 0.04**
**2**	Elbow	45	28	0.61	106	6.24	2.53	0.47 ± 0.08	0.43 ± 0.07	**-0.08 ± 0.02**
**3**	Dixie	50	20	0.39	88	5.18	2.48	0.44 ± 0.08	0.39 ± 0.07	**-0.10 ± 0.05**
**4**	Conch	47	18	0.37	85	5.00	2.47	0.51 ± 0.09	0.40 ± 0.06	**-0.23 ± 0.05**
**5**	Davis	47	22	0.46	102	6.00	2.56	0.48 ± 0.07	0.44 ± 0.07	-0.09 ± 0.02
**6**	Molasses	48	22	0.45	93	5.47	2.55	0.49 ± 0.07	0.43 ± 0.07	**-0.13 ± 0.01**
**7**	Alligator	45	19	0.41	90	5.29	2.55	0.45 ± 0.07	0.42 ± 0.06	-0.08 ± 0.05
**8**	Tennessee	46	29	0.62	102	6.00	2.51	0.41 ± 0.07	0.40 ± 0.07	-0.04 ± 0.04
**9**	Sprigger	32	18	0.55	73	4.29	2.49	0.44 ± 0.07	0.38 ± 0.06	**-0.17 ± 0.03**
**10**	Sluiceway	48	22	0.45	66	3.88	2.43	0.42 ± 0.07	0.34 ± 0.06	**-0.20 ± 0.04**
**11**	Marathon	22	10	0.43	67	3.94	2.45	0.44 ± 0.09	0.42 ± 0.05	0.08 ± 0.14
**12**	Pigeon	43	17	0.38	75	4.41	2.44	0.38 ± 0.06	0.33 ± 0.05	**-0.12 ± 0.05**
**13**	Bahia Honda	47	15	0.30	67	3.94	2.43	0.39 ± 0.08	0.35 ± 0.06	-0.09 ± 0.07
**14**	Water	39	12	0.29	67	3.94	2.47	0.42 ± 0.07	0.38 ± 0.05	-0.10 ± 0.08
**15**	Crane	31	12	0.37	59	3.47	2.37	0.29 ± 0.06	0.28 ± 0.05	-0.05 ± 0.08
**16**	Key West	23	2	0.05	35	2.06	2.03	0.41 ± 0.12	0.21 ± 0.06	-0.94 ± 0.04
**17**	Tampa Bay	33	6	0.16	47	2.76	2.24	0.21 ± 0.07	0.18 ± 0.05	-0.09 ± 0.08
**18**	Florida Bay	123	6	0.04	54	3.18	2.36	0.41 ± 0.08	0.34 ± 0.05	-0.19 ± 0.10
**19**	Bahamas	44	19	0.42	69	4.06	2.37	0.32 ± 0.08	0.29 ± 0.07	-0.08 ± 0.05
**20**	Bermuda	107	20	0.18	67	3.94	2.39	0.26 ± 0.06	0.29 ± 0.06	**0.12 ± 0.07**

Numeric codes are provided alongside location name for each population. Sample size (*N*), number of unique multilocus genotypes (*G*), genotypic richness (*R*), total number of alleles (*A*), average number of alleles per locus (*N*_*A*_), allelic richness per locus (*A*_*R*_), observed heterozygosity (*H*_*o*_), expected heterozygosity (*H*_*e*_) and inbreeding coefficient (*F*_*IS*_) are reported for each population. Standard error is included for *H*_*o*_, *H*_*e*_ and *F*_*IS*_. Values in bold indicate significant deviation from Hardy-Weinberg equilibrium at *p**-value* < 0.05.

### Genetic differentiation between populations

AMOVA revealed significant genetic differentiation between all populations (*F*_*ST*_ = 0.149 ± 0.017, *p-value* = 0.001) and significant genetic differentiation between the Gulf and Atlantic populations (*F*_*ST*_ = 0.109 ± 0.027, *p-value* = 0.001). The results of pairwise population differentiation were consistent across both statistics, *F*_*ST*_ and Jost’s *D* (S2 Table), with maximum values calculated as 0.531 and 0.295, respectively. Similar patterns in relative differentiation between populations were observed for both statistics, and differences were primarily in the magnitude of pairwise values, thus only *F*_*ST*_ will be described in detail. Pairwise differentiation values were low to moderate within Atlantic populations (*F*_*ST*_ = 0.000–0.092), and low to high within Gulf populations (*F*_*ST*_ = 0.012–0.237). Pairwise differentiation values between Atlantic and Gulf populations ranged from 0.041 between Davis in the Upper Keys and Marathon near the Middle Keys, to 0.330 between Conch in the Upper Keys and Crane in the Lower Keys. Florida Bay exhibited similar levels of differentiation between Gulf (*F*_*ST*_ = 0.144–0.273) and Atlantic (*F*_*ST*_ = 0.177–0.259) sites. Tampa Bay, the westernmost site sampled, exhibited high differentiation between Atlantic populations (*F*_*ST*_ = 0.236–0.37) and moderate to high differentiation between Gulf populations (*F*_*ST*_ = 0.101–0.279). The highest overall pairwise differentiation was found between the Bahamas and Tampa Bay, where *F*_*ST*_ = 0.531. The next greatest values were found between the Bahamas and Gulf populations (*F*_*ST*_ = 0.261–0.473), and values were moderate between the Bahamas and Atlantic populations (*F*_*ST*_ = 0.192 0.259). Bermuda pairwise differentiation with Atlantic and Gulf sites was moderate to high, with *F*_*ST*_ values ranging from 0.142 to 0.224 and 0.176 to 0.280, respectively.

In the PCA, the first two principal component axes contained 18.3% and 7.7% of total variance, respectively (S1 Fig). The Atlantic and Gulf sites clustered separately, with some overlap occurring, mostly between Gulf sites proximal to breaks in the Middle keys (Marathon, Pigeon and Sprigger), and Atlantic sites. Tampa Bay clustered with the Gulf sites, while Bermuda clustered between Gulf and Atlantic sites. The Bahamas clustered separately from all other sites.

### Population structure and gene flow

**Fig 2:**
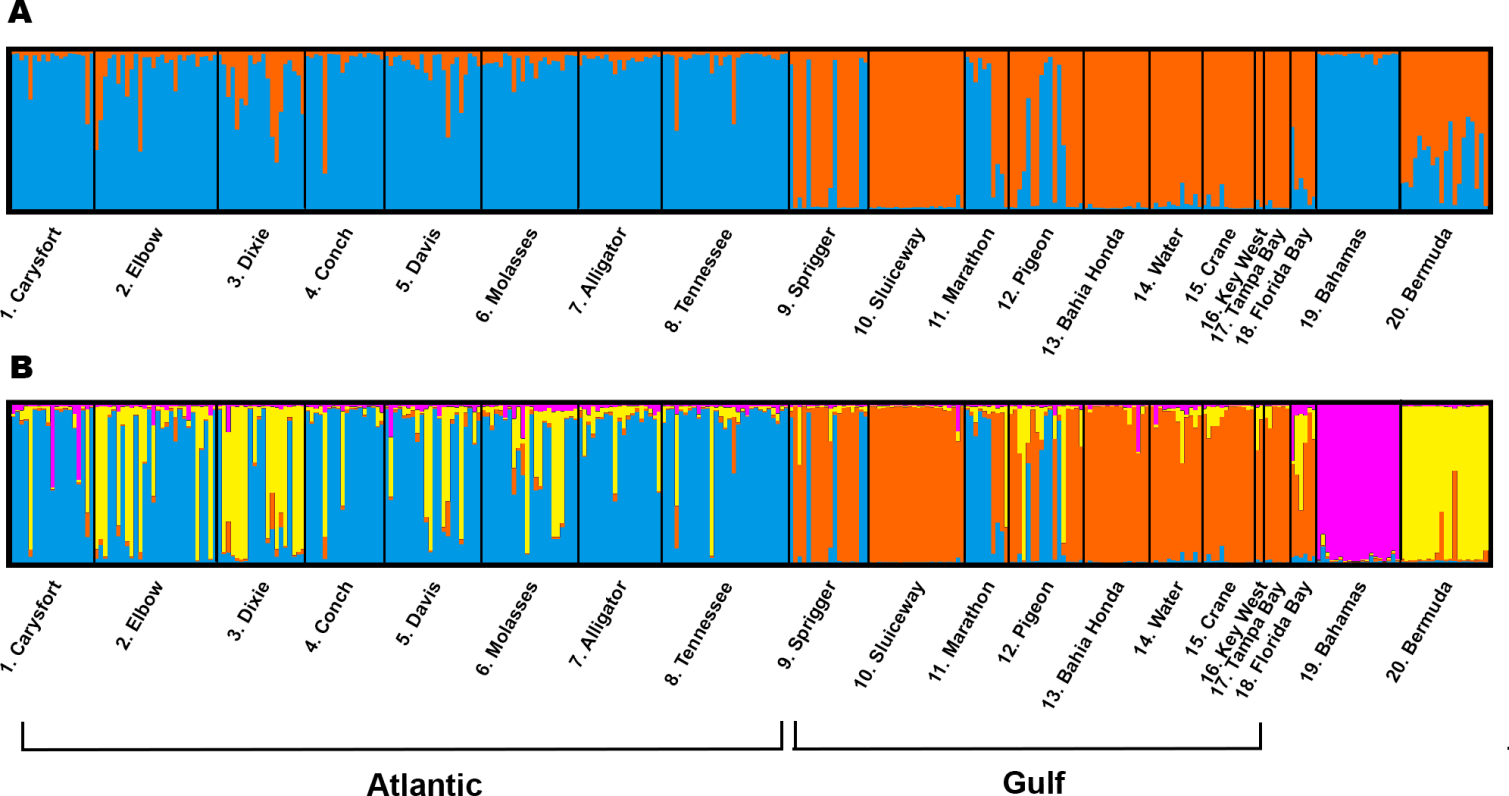
Diagrams of STRUCTURE cluster assignment. (A) K = 2 cluster assignment and (B) K = 4 cluster assignment. Population names are on the x-axis, separated by black vertical bands. Individual genotypes are represented as vertical bars and cluster assignment is depicted by color.

Population structure was present, with greatest statistical support for K = 2 (Δ*K* = 297.26), followed by K = 4 (Δ*K* = 20.84) number of population clusters. For K = 2, Atlantic and Gulf populations clustered separately, and Tampa Bay, Florida Bay and the Bahamas were assigned to the Gulf cluster (Fig 2). The genotypes in the Bermuda population show mixed membership to both the Atlantic and Gulf clusters. For K = 4, the Atlantic and Gulf populations still clustered separately, and the Bahamas and Bermuda were assigned to distinct clusters. For both K = 2 and K = 4, Gulf sites proximal to breaks in the Middle Keys (Sprigger, Marathon and Pigeon) exhibit admixture with Atlantic populations.

**Fig 3:**
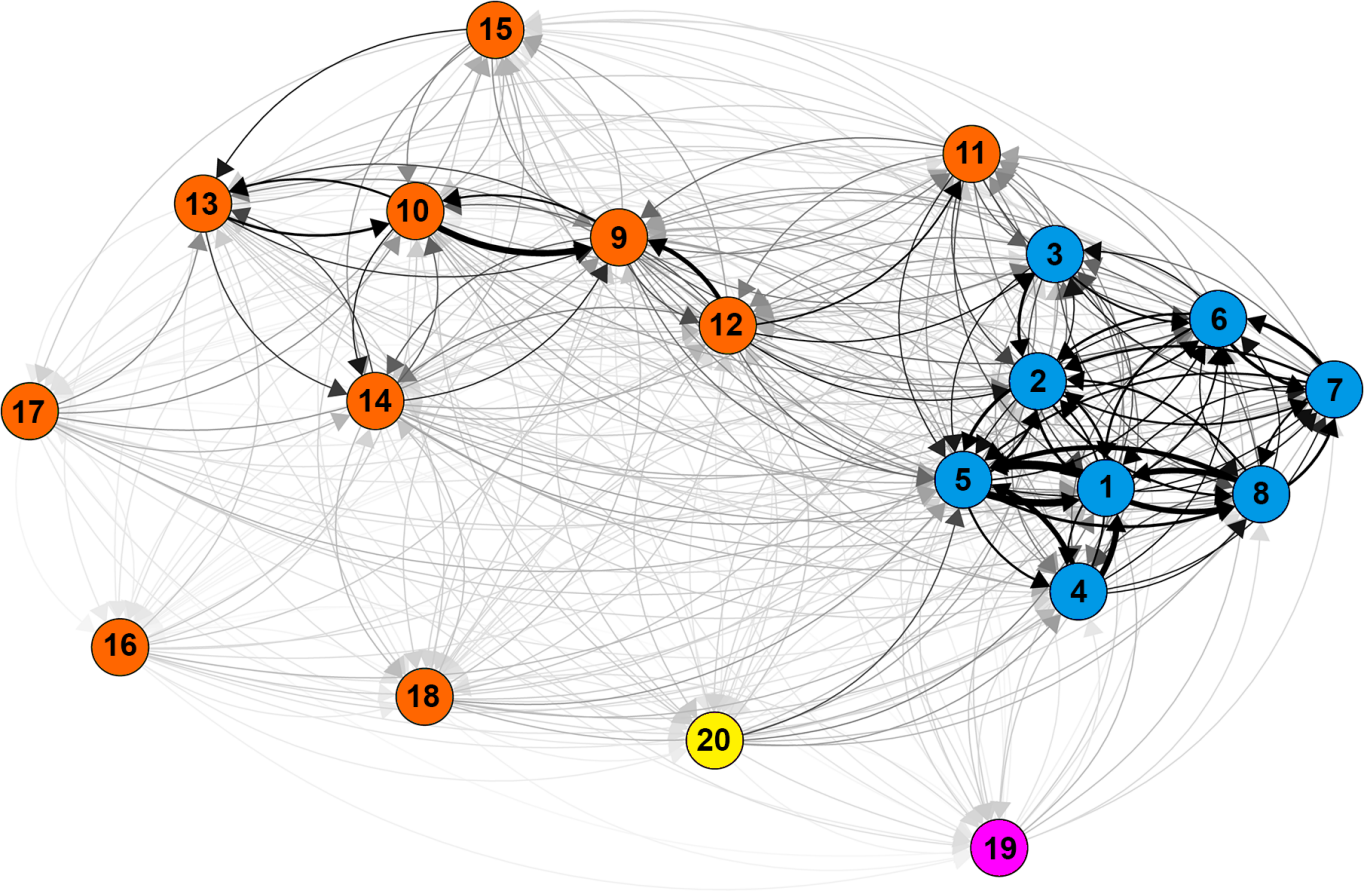
Diagram of relative magnitude and direction of gene flow. Nodes represent populations (refer to Table 1 to match numeric codes with location names). Arrows are weighted according to Alcala's Nm values (S3 Table), which range from 0.004 to 1.000, and arrowheads show the estimated direction of gene flow.

Average migration between all populations was 1.7 and 2.6 migrants per generation, following the *F*_*ST*_ method and private alleles method, respectively. Relative pairwise migration was highest among Atlantic populations, ranging from 0.174 to 1, on a scale from 0 to 1 (Fig 3; S3 Table). Within the Atlantic group, lowest genetic exchange occurred from Conch to Elbow, and the highest from Davis to Carysfort. Exchange within the Gulf populations (excluding Key West) ranged from 0.029 to 0.792, and the greatest exchange occurred between Sluiceway and Sprigger, both located on the western edge of Florida Bay. Exchange to and from Key West was particularly low and did not exceed 0.084. There was greater relative migration from Atlantic sites to Gulf sites proximal to a break in the Middle Keys (Marathon, Pigeon and Sprigger) than there was from within the Gulf. Florida Bay exhibited relative migration rates lower than 0.125 with greatest outgoing migration to the Atlantic site Davis. Tampa Bay exhibited migration rates lower than 0.148 with highest migration coming from Gulf sites. The Bahamas exhibited negligible migration rates, not exceeding 0.085. Incoming relative migration to Bermuda was always less than 0.067, while outgoing migration ranged from 0.01 to 0.23, with the highest rates of exchange occurring with the Atlantic populations.

## Discussion

Within the Florida Keys and subtropical Atlantic region, *S*. *filiforme* exhibits low genetic diversity when compared with other temperate and tropical seagrass species. We found 1) the level of clonality in *S*. *filiforme*, as measured by shared multilocus genotypes, to be highly variable among populations; 2) low allelic diversity and heterozygote excess in almost every population; 3) evidence of genetic differentiation and population structure, in which the sampled populations were assigned to two major demes separated by the Florida Keys archipelago; and 4) asymmetric gene flow patterns, though average migration rates across all populations exceeded one migrant per generation.

Genotypic richness of *S*. *filiforme* varied widely across sampling sites, though this cannot be completely explained by disparities in sampling area. Sample collections in 2014 were from within an area of ~ 2,500 m^2^, in which genotypic richness ranged from 0.37 to 0.62. Sample collections in 2015 were from within an area of ~ 500 m^2^, in which genotypic richness ranged from 0.05 to 0.43. Therefore, within each sampling scheme, we observed a wide range in clonality. In the larger areas sampled in 2014, we detected one genet present in two adjacent populations (Sprigger and Sluiceway), extending over hundreds of meters. For the 2015 collection sites, sampling in a smaller total area with shorter distances between each shoot sampled may have led to a decrease in detection efficiency of total genets and number of alleles present in the population [68]. Allelic richness standardized by smallest sample size was consistent across all sites, suggesting the observed higher number of alleles and unique MLGs in 2014 collection populations were related to the spatial scale of sampling and do not necessarily indicate greater diversity in the Atlantic populations. Low genotypic richness in some populations and low allelic diversity in all populations of *S*. *filiforme* across the subtropical Atlantic region underscores the advantage of clonal reproduction in this environment. It is also possible that we observed an edge-of-range effect [69] in which populations of a species closer toward their range limit express lower genetic diversity than populations in the center of the species' range. Though our study did not span across the center of distribution for *S*. *filiforme*, we would expect to find greater levels of diversity in Caribbean populations.

The strongest population structure clearly develops in South Florida, where Tampa Bay, though hundreds of kilometers away from the Florida Keys, groups with Gulf populations, suggesting the Florida Keys archipelago presented historical barriers to gene flow between Gulf and Atlantic demes, and perhaps continues to impede gene flow with contemporary land configurations and sea levels. This is also supported by the relative migration and direction of gene flow calculations, which revealed asymmetric patterns in the magnitude and direction of genetic exchange. The Atlantic populations are strongly connected to one another, as are the Gulf populations, though to a lesser extent. The genetic disjunction between Atlantic and Gulf *S*. *filiforme* populations in Florida may provide evidence of a phylogeographic break, which has been observed for a number of warm-temperate marine and intertidal organisms, related to increases in seawater temperature (and thereby northward shifts in temperate species’ range limits) associated with glacial retreats that occurred throughout the Pleistocene [70–73].

Historical changes in sea level (and not necessarily temperature) may have been a primary factor contributing to the development of the genetic break for *S*. *filiforme*, a tropical species tolerant of warm seawater temperatures. During the Pleistocene, glacial advances exposed more of the Florida peninsula and may have restricted estuarine habitat to a small area within the western Gulf of Mexico [74], while glacial retreats increased sea level and promoted the expansion of estuarine habitat, likely causing increased contact between eurythermal species along the southern tip of the peninsula [75]. Depending on Pliocene distributions of *S*. *filiforme*, changes in sea level that resulted in the final emergence of the Florida peninsula may have instigated the genetic break. McCommas [76] attributed the genetic discontinuity between the Gulf of Mexico and the Atlantic populations of the sea anemone, *Bunodosoma carvernata*, to this vicariant event based on estimated time since divergence. Without fossil evidence or molecular clock calculations, we can merely suggest the break we found in Florida was similarly initiated by prior fluctuations in sea level and maintained by contemporary ocean currents.

Interestingly, there are exceptions to the Atlantic-Gulf divide for *S*. *filiforme*, in which we detected relatively high gene flow from Atlantic populations to Gulf populations proximal to a break in the Middle Keys (at sites Marathon, Pigeon and Sprigger). Additionally, the Marathon population appeared more genetically similar to the Atlantic populations than to those in Gulf. This finding could reflect shared ancestry and relatively recent divergence between the Atlantic and Gulf populations, but does not exclude the possibility of genetic exchange occurring between Atlantic and Gulf populations across the archipelago via propagules or rafting vegetation.

We found relative gene flow between proximal Florida Bay, Tampa Bay and Key West populations and other South Florida populations to be comparable to (and in some instances less than) gene flow levels observed between Florida and more distant non-Florida populations. These populations were also highly clonal, exhibiting the lowest genotypic richness values measured in this study. We sampled in the northeastern-most extent of Florida Bay in Blackwater Sound, an enclosed area with few hydrological connections to the greater Florida Bay or the Atlantic; we suspect the low gene flow and low genotypic richness are related to this isolation. Gene flow between the Key West population and other South Florida populations may be limited by hydrologic rather than topographic isolation: strong reversing tidal currents flowing between the Gulf of Mexico and the Florida Straits may prevent mixing between the Key West population and the further eastward Lower Keys populations. The Tampa Bay population, located roughly halfway up the Florida peninsula, approaches the northern range limit for *S*. *filiforme* in the Gulf of Mexico. In the latter half of the 20^th^ century, Tampa Bay experienced a major decline (~ 70%) in historical seagrass coverage due to rapid population expansion and development along the coast [77]. Since adopting policies to prevent pollution and dredging activities, seagrasses in Tampa Bay have been on a recovery trajectory and now exceed historical extent [78]. It is unclear whether the low gene flow and high clonality in this population reflects its northern position, past seagrass decline or contemporary dispersal limitations. The Tampa Bay population is not representative of the entire estuary as the samples were collected near the mouth of the bay, disregarding the meadows within the interior of the bay. Further research on the population genetics of all seagrass species throughout Tampa Bay is warranted, particularly given its tumultuous history of environmental decline, restoration and recovery.

Though Bermuda is the furthest distance from all other populations, the greatest differentiation occurred between the Bahamas and Florida sites. High relatedness between Florida and Bermuda populations of *H*. *wrightii* [79] indicates similar mechanisms may be responsible for this pattern. Population structure and gene flow patterns in the Bahamas and Bermuda populations were somewhat counter to our expectations, but must be interpreted with caution because we only sampled one site from each location. In the Δ*K* = 2 population clusters scenario, the Bermuda population contains genotypes with near equal membership to the Atlantic and Gulf clusters, while the Bahamas population shows complete membership to the Atlantic cluster. These results imply the Bahamas population groups with the Atlantic populations, though our previous analyses suggest relatively strong genetic differentiation and limited gene flow between the Bahamas and all other sites. Contemporary surface ocean currents directing the movement of propagules, and therefore genetic exchange between populations, may be responsible for these patterns [80]. Based on the mixed-membership genotypes in the Bermuda population, it is plausible the Bermuda population developed from an initial source population in recent evolutionary history that later diverged to create the two major clusters identified here, and now receives propagules from Florida via the Gulf Stream at a frequency sufficient to prevent strong genetic differentiation. The moderate degree of gene flow from Bermuda to the Florida populations estimated here (Fig 3) is interesting, as propagule dispersal via surface currents in the opposite direction (South to North) along the Gulf Stream seems more likely. And despite the westward flow of the Antilles current, the topography of the islands of the Bahamas might restrict gene flow between Florida populations and the remote sampling location in San Salvador, the easternmost island of the Bahamas. We believe further sampling across the subtropical Atlantic, especially along the western Bahamian islands, will clarify unexpected gene flow patterns.

The high genetic exchange within Atlantic populations as evidenced by high migration rates may be explained by hydrologic connections created by surface currents and eddies that form along the Florida Keys Atlantic coastline. The gene flow patterns observed here roughly agree with the modeled and observed movement of spiny lobster (*Panulirus argus*) larvae along a ‘recruitment conveyor’ in the Florida Keys, in which spawning larvae near the Yucatán Peninsula have been identified as source populations [81]. The net eastward and northward movement of the Florida current along the Florida Shelf and the intermittent formation of small eddies could facilitate local movement and entrainment of seagrass propagules [82]. Less genetic exchange within the Gulf populations is perhaps related to the isolating topography of the Lower Keys, in which several small key islands and narrow channels separate the seagrass meadows, potentially hindering the movement of propagules. Though mean hydrological transport occurs from the Gulf to the Atlantic, westward tidal flow sometimes pushes Atlantic waters through channels in the Keys [83,84] and could promote movement of propagules of Atlantic origin through to Gulf side populations Marathon, Pigeon and Sprigger, facilitating the admixture of genotypes detected between clusters.

The population genetics of *S*. *filiforme* in the subtropical Atlantic appear to match theoretical predictions for a colonizer species. The *S*. *filiforme* meadows we sampled contained variable genotypic diversity, likely a result of site-specific properties influencing the growth and reproductive strategies in this species as well as propagule supply [85]. The low allelic diversity within *S*. *filiforme* meadows and evidence for population structure along the possible phylogeographic boundary in the Florida Keys, are typical of colonizers. These findings are consistent with a study on the only congener of *S*. *filiforme*, *Syringodium isoetifolium*, which also exhibited variable genotypic diversity and population structure defined by bioregions in the western North Pacific [86]. The climax species of the tropical and subtropical Atlantic, *T*. *testudinum*, exhibited high genotypic and allelic diversity, and no evidence of population structure in Florida Bay [37], and similarly high allelic diversity and little evidence of population structure across ~ 1000 km of coastline along the Yucatan Peninsula in Mexico [87]. In contrast, the colonizer species *H*. *wrightii* showed high clonality and strong differentiation among edge-of-range populations in Florida, North Carolina and Bermuda [79], and generally high clonality and weak population structure along the western Gulf of Mexico coast [88]. The population genetics of *S*. *filiforme* conform to expectations for colonizer species, with genotypic diversity mediated by local conditions and meadow demographics.

Successional status is derived from environmental tolerances and growth and reproductive strategies that in turn impact population genetics, while modern oceanic hydrology ultimately controls dispersal trajectories and therefore genetic exchange. It is likely that evolutionarily historical population dynamics under past continent arrangements and sea levels are the dominant forces driving the population structure in *S*. *filiforme* in the subtropical Atlantic Ocean. The higher genotypic diversity found in *S*. *filiforme* in certain populations suggests that some meadows may be more resilient to disturbances than others, and these more resilient meadows may enhance recovery of depauperate meadows by sustaining a supply of propagules and gene flow, but only where ocean currents and land barriers do not impede connectivity. Whether overall low genetic diversity and strong population structure in subtropical Atlantic populations of *S*. *filiforme* equates to limited capability for adaptation to selective pressures has yet to be tested.

## Supporting information

S1 Table Sample site GPS coordinates. GPS coordinates mark the exact location of each sample site. Latitude and longitude are in decimal degrees.
(DOCX)

S2 Table Pairwise genetic differentiation. *F*_*ST*_ values are provided to the left of the diagonal and Jost's D values are provided to the right of the diagonal. Bold text indicates significance based on non-overlapping confidence intervals.
(DOCX)

S3 Table Values for relative magnitude and direction of gene flow. Values represent the relative amount of gene flow from populations in the first column to receiving populations identified in the first row. For example, the highest amount of gene flow (1.000) occurs from Carysfort to Davis, while the lowest amount of gene flow occurs from the Bahamas to Key West (0.004). Bold text indicate significance based on non-overlapping 95% confidence intervals.
(DOCX)

S1 Fig Principal components analysis (PCA) plot. Axis loading values are depicted for the two principle coordinate axes containing the greatest amount of variation, PC1 (18.3% variance) and PC2 (7.7% variance). Genotypes from each population group are distinguished by color and shape (Atlantic: blue circles, Gulf: orange triangles, Bermuda: yellow diamonds, Bahamas: magenta squares).
(EPS)
