# Supplementary material for: Population structure and gene flow of the tropical seagrass, Syringodium filiforme, in the Florida Keys and subtropical Atlantic region
Source: PLoS One. 2018 Sep 5;13(9):e0203644. doi: 10.1371/journal.pone.0203644 (PMC6124813; doi:10.1371/journal.pone.0203644)
Supplement: S1 Table — GPS coordinates mark the exact location of each sample site. Latitude and longitude are in decimal degrees. (DOCX) [file pone.0203644.s001.docx]

| **Population** | | **Longitude** | **Latitude** |
| --- | --- | --- | --- |
| **1** | Carysfort | -80.235833 | 25.212917 |
| **2** | Elbow | -80.295425 | 25.15691 |
| **3** | Dixie | -80.339417 | 25.072783 |
| **4** | Conch | -80.404084 | 25.027581 |
| **5** | Davis | -80.456617 | 24.966544 |
| **6** | Molasses | -80.507069 | 24.930162 |
| **7** | Alligator | -80.619423 | 24.86796 |
| **8** | Tennessee | -80.759673 | 24.776892 |
| **9** | Sprigger | -80.940355 | 24.912795 |
| **10** | Sluiceway | -80.98605 | 24.8964 |
| **11** | Marathon | -81.152833 | 24.8152 |
| **12** | Pigeon | -81.169267 | 24.75525 |
| **13** | Bahia Honda | -81.287683 | 24.788883 |
| **14** | Water | -81.444783 | 24.767283 |
| **15** | Crane | -81.5505 | 24.766633 |
| **16** | Key West | -81.846617 | 24.5505 |
| **17** | Tampa Bay | -82.675241 | 27.653497 |
| **18** | Florida Bay | -80.408641 | 25.145837 |
| **19** | Bahamas | -74.457861 | 24.124222 |
| **20** | Bermuda | -64.723871 | 32.34918 |
