# Supplementary material for: Population structure and gene flow of the tropical seagrass, Syringodium filiforme, in the Florida Keys and subtropical Atlantic region
Source: PLoS One. 2018 Sep 5;13(9):e0203644. doi: 10.1371/journal.pone.0203644 (PMC6124813; doi:10.1371/journal.pone.0203644)
Supplement: S2 Table — FST values are provided to the left of the diagonal and Jost's D values are provided to the right of the diagonal. Bold text indicates significance based on non-overlapping confidence intervals. (DOCX) [file pone.0203644.s002.docx]

|  |  | **1** | **2** | **3** | **4** | **5** | **6** | **7** | **8** | **9** | **10** | **11** | **12** | **13** | **14** | **15** | **16** | **17** | **18** | **19** | **20** |
| --- | --- | --- | --- | --- | --- | --- | --- | --- | --- | --- | --- | --- | --- | --- | --- | --- | --- | --- | --- | --- | --- |
| **1** | Carysfort |  | 0.0093 | **0.0229** | 0.0004 | 0.0000 | 0.0042 | 0.0095 | 0.0001 | **0.0568** | **0.0974** | 0.0134 | **0.0620** | **0.1283** | **0.0872** | **0.1931** | **0.0786** | **0.1927** | **0.0999** | **0.0732** | **0.0629** |
| **2** | Elbow | **0.0279** |  | 0.0064 | **0.0236** | 0.0092 | 0.0082 | 0.0049 | **0.0119** | **0.0344** | **0.0686** | 0.0145 | **0.0305** | **0.0806** | **0.0608** | **0.1171** | **0.0268** | **0.1370** | **0.0889** | **0.0965** | **0.0433** |
| **3** | Dixie | **0.0608** | **0.0211** |  | **0.0265** | **0.0266** | 0.0064 | 0.0133 | **0.0278** | **0.0192** | **0.0406** | 0.0087 | **0.0170** | **0.0568** | **0.0530** | **0.0827** | **0.0301** | **0.0815** | **0.0599** | **0.1107** | **0.0275** |
| **4** | Conch | 0.0132 | **0.0556** | **0.0922** |  | 0.0040 | **0.0175** | **0.0231** | **0.0104** | **0.0696** | **0.1182** | 0.0115 | **0.0634** | **0.1425** | **0.1127** | **0.2015** | **0.0936** | **0.2066** | **0.0951** | **0.0807** | **0.0669** |
| **5** | Davis | -0.0004 | **0.0250** | **0.0604** | 0.0165 |  | 0.0085 | 0.0077 | 0.0041 | **0.0512** | **0.0913** | 0.0120 | **0.0454** | **0.1095** | **0.0662** | **0.1583** | **0.0427** | **0.1747** | **0.0824** | **0.0814** | **0.0569** |
| **6** | Molasses | **0.0337** | **0.0318** | **0.0320** | **0.0593** | **0.0465** |  | 0.0010 | **0.0150** | **0.0260** | **0.0526** | 0.0198 | **0.0306** | **0.0854** | **0.0588** | **0.1232** | **0.0551** | **0.1231** | **0.0635** | **0.0862** | **0.0640** |
| **7** | Alligator | **0.0365** | 0.0202 | **0.0446** | **0.0613** | **0.0348** | 0.0114 |  | 0.0055 | **0.0317** | **0.0614** | **0.0220** | **0.0298** | **0.0784** | **0.0367** | **0.1226** | **0.0629** | **0.1295** | **0.0816** | **0.0951** | **0.0774** |
| **8** | Tennessee | **0.0042** | **0.0267** | **0.0658** | **0.0440** | **0.0201** | **0.0455** | **0.0278** |  | **0.0562** | **0.0931** | 0.0204 | **0.0574** | **0.1191** | **0.0822** | **0.1760** | **0.0872** | **0.1841** | **0.1342** | **0.0915** | **0.0699** |
| **9** | Sprigger | **0.1368** | **0.0850** | **0.0715** | **0.1500** | **0.1071** | **0.0909** | **0.1025** | **0.1404** |  | 0.0000 | 0.0141 | 0.0012 | 0.0094 | 0.0058 | **0.0230** | 0.0000 | **0.0207** | **0.0521** | **0.1590** | **0.0471** |
| **10** | Sluiceway | **0.2137** | **0.1525** | **0.1283** | **0.2409** | **0.1798** | **0.1533** | **0.1688** | **0.2086** | 0.0117 |  | **0.0445** | **0.0135** | 0.0031 | 0.0042 | **0.0285** | 0.0009 | **0.0234** | **0.0708** | **0.1848** | **0.0601** |
| **11** | Marathon | **0.0512** | **0.0531** | **0.0454** | **0.0519** | **0.0414** | **0.0566** | **0.0717** | **0.0759** | **0.0692** | **0.1291** |  | 0.0048 | **0.0709** | **0.0281** | **0.0898** | **0.0355** | **0.1003** | **0.0387** | **0.1035** | **0.0378** |
| **12** | Pigeon | **0.1529** | **0.0838** | **0.0622** | **0.1605** | **0.1240** | **0.1036** | **0.1108** | **0.1565** | **0.0361** | **0.0895** | 0.0460 |  | **0.0337** | **0.0190** | **0.0226** | 0.0041 | **0.0265** | **0.0530** | **0.1228** | **0.0333** |
| **13** | Bahia Honda | **0.2377** | **0.1775** | **0.1658** | **0.2623** | **0.1939** | **0.1950** | **0.1856** | **0.2349** | 0.0516 | 0.0327 | **0.1667** | **0.1344** |  | 0.0070 | 0.0133 | 0.0007 | **0.0367** | **0.1045** | **0.2235** | **0.0865** |
| **14** | Water | **0.1846** | **0.1259** | **0.1338** | **0.2152** | **0.1455** | **0.1437** | **0.1261** | **0.1872** | **0.0477** | **0.0539** | **0.1196** | **0.1065** | 0.0480 |  | **0.0337** | 0.0066 | **0.0726** | **0.0402** | **0.1429** | **0.0735** |
| **15** | Crane | **0.2966** | **0.2123** | **0.1992** | **0.3295** | **0.2557** | **0.2371** | **0.2345** | **0.2975** | **0.1118** | **0.1246** | **0.2367** | **0.1543** | **0.0750** | **0.1338** |  | 0.0022 | 0.0115 | **0.0726** | **0.2480** | **0.0806** |
| **16** | Key West | **0.2001** | **0.1140** | **0.1145** | **0.2327** | **0.1517** | **0.1588** | **0.1639** | **0.1945** | **0.0368** | **0.0673** | **0.1208** | **0.0992** | **0.0691** | **0.0994** | **0.1003** |  | 0.0096 | **0.0506** | **0.2057** | **0.0454** |
| **17** | Tampa Bay | **0.3263** | **0.2468** | **0.2357** | **0.3650** | **0.3019** | **0.2535** | **0.2713** | **0.3295** | **0.1271** | **0.1593** | **0.2791** | **0.2171** | **0.1891** | **0.2272** | **0.1012** | **0.2592** |  | **0.0977** | **0.2950** | **0.1169** |
| **18** | Florida Bay | **0.2119** | **0.1819** | **0.1783** | **0.2185** | **0.1832** | **0.1769** | **0.1954** | **0.2585** | **0.1774** | **0.2231** | **0.1435** | **0.1891** | **0.2366** | **0.1559** | **0.2733** | **0.2225** | **0.3358** |  | **0.1373** | **0.0813** |
| **19** | Bahamas | **0.1918** | **0.2162** | **0.2587** | **0.2366** | **0.2123** | **0.2039** | **0.2240** | **0.2337** | **0.3107** | **0.3699** | **0.2614** | **0.3145** | **0.3963** | **0.3159** | **0.4734** | **0.4533** | **0.5305** | **0.3713** |  | **0.1183** |
| **20** | Bermuda | **0.1966** | **0.1399** | **0.1418** | **0.2244** | **0.1730** | **0.1812** | **0.1980** | **0.2051** | **0.1887** | **0.2340** | **0.1854** | **0.1756** | **0.2521** | **0.2261** | **0.2799** | **0.2320** | **0.3686** | **0.2685** | **0.3563** |  |
