# Supplementary material for: Population structure and gene flow of the tropical seagrass, Syringodium filiforme, in the Florida Keys and subtropical Atlantic region
Source: PLoS One. 2018 Sep 5;13(9):e0203644. doi: 10.1371/journal.pone.0203644 (PMC6124813; doi:10.1371/journal.pone.0203644)
Supplement: S3 Table — Values represent the relative amount of gene flow from populations in the first column to receiving populations identified in the first row. For example, the highest amount of gene flow (1.000) occurs from Carysfort to Davis, while the lowest amount of gene flow occurs from the Bahamas to Key West (0.004). Bold text indicate significance based on non-overlapping 95% confidence intervals. (DOCX) [file pone.0203644.s003.docx]

|  |  | **1** | **2** | **3** | **4** | **5** | **6** | **7** | **8** | **9** | **10** | **11** | **12** | **13** | **14** | **15** | **16** | **17** | **18** | **19** | **20** |
| --- | --- | --- | --- | --- | --- | --- | --- | --- | --- | --- | --- | --- | --- | --- | --- | --- | --- | --- | --- | --- | --- |
| **1** | Carysfort |  | 0.451 | 0.230 | 0.386 | 1.000 | 0.369 | 0.311 | 0.722 | 0.095 | 0.027 | 0.138 | 0.089 | 0.028 | 0.034 | 0.029 | 0.012 | 0.012 | 0.029 | 0.065 | 0.030 |
| **2** | Elbow | 0.467 |  | 0.306 | 0.171 | 0.522 | 0.392 | 0.389 | 0.417 | 0.110 | 0.040 | 0.118 | 0.108 | 0.042 | 0.055 | 0.045 | 0.019 | 0.020 | 0.046 | 0.052 | 0.048 |
| **3** | Dixie | 0.237 | 0.487 |  | 0.119 | 0.299 | 0.400 | 0.293 | 0.231 | 0.123 | 0.059 | 0.135 | 0.148 | 0.060 | 0.062 | 0.053 | 0.019 | 0.024 | 0.054 | 0.038 | 0.058 |
| **4** | Conch | 0.769 | 0.382 | 0.174 |  | 0.671 | 0.331 | 0.282 | 0.305 | 0.111 | 0.024 | 0.158 | 0.093 | 0.025 | 0.030 | 0.024 | 0.011 | 0.010 | 0.024 | 0.039 | 0.021 |
| **5** | Davis | 0.876 | 0.457 | 0.192 | 0.426 |  | 0.354 | 0.311 | 0.479 | 0.124 | 0.034 | 0.146 | 0.100 | 0.035 | 0.045 | 0.031 | 0.014 | 0.013 | 0.029 | 0.049 | 0.034 |
| **6** | Molasses | 0.428 | 0.432 | 0.322 | 0.198 | 0.312 |  | 0.533 | 0.309 | 0.123 | 0.043 | 0.115 | 0.130 | 0.036 | 0.046 | 0.042 | 0.013 | 0.018 | 0.046 | 0.058 | 0.042 |
| **7** | Alligator | 0.350 | 0.439 | 0.184 | 0.177 | 0.411 | 0.590 |  | 0.398 | 0.112 | 0.032 | 0.103 | 0.130 | 0.031 | 0.036 | 0.038 | 0.013 | 0.015 | 0.038 | 0.054 | 0.036 |
| **8** | Tennessee | 0.747 | 0.424 | 0.200 | 0.232 | 0.661 | 0.308 | 0.465 |  | 0.089 | 0.026 | 0.118 | 0.090 | 0.026 | 0.034 | 0.030 | 0.012 | 0.013 | 0.027 | 0.061 | 0.030 |
| **9** | Sprigger | 0.145 | 0.174 | 0.172 | 0.104 | 0.184 | 0.208 | 0.113 | 0.129 |  | 0.397 | 0.169 | 0.232 | 0.240 | 0.208 | 0.099 | 0.034 | 0.037 | 0.058 | **0.028** | 0.061 |
| **10** | Sluiceway | **0.089** | **0.072** | 0.068 | **0.057** | **0.104** | **0.118** | **0.071** | **0.078** | 0.792 |  | 0.115 | 0.093 | 0.407 | 0.299 | 0.095 | 0.029 | 0.031 | 0.059 | **0.043** | 0.038 |
| **11** | Marathon | 0.232 | 0.244 | 0.211 | 0.132 | 0.278 | 0.236 | 0.167 | 0.165 | 0.198 | 0.061 |  | 0.168 | 0.061 | 0.068 | 0.041 | 0.018 | 0.018 | 0.048 | 0.025 | 0.031 |
| **12** | Pigeon | 0.139 | **0.304** | 0.339 | 0.094 | 0.197 | 0.162 | 0.098 | 0.116 | 0.609 | 0.208 | 0.368 |  | 0.163 | 0.171 | 0.114 | 0.028 | 0.037 | 0.053 | 0.025 | 0.067 |
| **13** | Bahia Honda | **0.052** | 0.037 | 0.035 | 0.034 | 0.065 | 0.048 | 0.049 | **0.050** | 0.268 | 0.443 | 0.057 | 0.065 |  | 0.256 | 0.103 | 0.025 | 0.023 | 0.035 | 0.023 | 0.038 |
| **14** | Water | **0.089** | 0.084 | 0.067 | 0.057 | **0.119** | **0.102** | **0.111** | **0.093** | 0.274 | 0.208 | 0.103 | 0.094 | 0.242 |  | 0.098 | 0.022 | 0.020 | 0.085 | 0.036 | 0.037 |
| **15** | Crane | **0.063** | 0.045 | 0.045 | 0.029 | **0.096** | 0.055 | 0.044 | 0.051 | 0.146 | 0.152 | 0.042 | 0.072 | 0.297 | 0.136 |  | 0.024 | 0.059 | 0.031 | 0.009 | **0.063** |
| **16** | Key West | **0.051** | **0.084** | **0.040** | **0.030** | **0.057** | **0.034** | **0.035** | **0.032** | **0.064** | **0.065** | **0.045** | 0.043 | **0.065** | **0.056** | 0.032 |  | **0.020** | **0.024** | **0.017** | **0.022** |
| **17** | Tampa Bay | **0.064** | 0.039 | 0.033 | **0.033** | **0.094** | **0.054** | **0.047** | **0.051** | **0.135** | **0.136** | 0.027 | 0.041 | **0.148** | **0.094** | 0.108 | 0.011 |  | 0.025 | **0.010** | 0.027 |
| **18** | Florida Bay | **0.091** | 0.063 | 0.050 | **0.090** | **0.125** | 0.038 | 0.067 | 0.046 | 0.074 | 0.051 | 0.070 | 0.060 | 0.063 | 0.074 | 0.035 | 0.013 | 0.016 |  | 0.013 | 0.018 |
| **19** | Bahamas | 0.085 | 0.057 | 0.035 | 0.029 | 0.031 | 0.070 | 0.046 | 0.044 | 0.017 | 0.019 | 0.015 | 0.017 | 0.022 | 0.039 | 0.012 | 0.004 | 0.005 | **0.039** |  | 0.010 |
| **20** | Bermuda | **0.161** | **0.119** | 0.082 | **0.125** | **0.234** | 0.047 | **0.075** | **0.092** | 0.045 | 0.025 | 0.027 | 0.066 | 0.026 | 0.052 | 0.027 | 0.011 | 0.013 | 0.027 | **0.017** |  |
